# Perceived Discrimination and Stigma in the Context of the Long-Term Care Insurance Law from the Perspectives of Arabs and the Jews in the North of Israel

**DOI:** 10.3390/ijerph16193511

**Published:** 2019-09-20

**Authors:** Liat Ayalon

**Affiliations:** School of Social Work, Bar-Ilan University, Ramat Gan 5290002, Israel; liat.ayalon@biu.ac.il; Tel.: +972-3-5317271

**Keywords:** home care, discrimination, racism, religion, Muslim, Arab, Jewish

## Abstract

The Long-Term Care Insurance Law provides support to older Israelis who wish to remain in their home. The present study evaluated the experience of perceived discrimination and stigma in the context of the law among Arab older adults, their family members, and their paid home care workers. For triangulation purposes, we interviewed 15 National Insurance Institute workers (NII; responsible for implementing the law; 47% Arab), 31 older adults (81% Arab), 31 family members (87% Arab), and six paid home care workers (83% Arab) in the north of Israel. Respondents were queried about their home care experience and their encounter with the NII. Thematic analysis was conducted. Four main themes emerged: (a) a strong sense of perceived discrimination among Arab interviewees, (b) reports suggesting the internalization of stigma and the adoption of negative views regarding the Arab population by some Arab respondents, (c) implicit stigma manifested in claims concerning the Arab population (primarily) as “cheating” the system, and (d) the negation of discrimination of Arabs as reported by Jewish interviewees and NII workers. The findings show that a sense of perceived discrimination is common and colors the experience of service seeking among Arabs. On the other hand, the Jewish interviewees in this study completely negated any discrimination or stigma directed toward Arabs. The findings point to the importance of group affiliation (e.g., minority vs. majority) in interpreting the existence of discrimination. The findings likely have major implications for both service providers and policy-makers and legislators.

## 1. Introduction

Discrimination is defined as the differential treatment of people or groups of people on the basis of arbitrary characteristics, such as gender, race, or age. Discrimination can be both positive and negative [1]. Moreover, discrimination can be explicit, directly expressed and manifested, or implicit, with the person performing the act of discrimination not even being aware of it [2]. Discrimination can occur at the individual level, as it is often manifested in everyday life in varied interpersonal contexts, or at the institutional level, as manifested in policies or legislations, which differentially treat certain groups [3]. Discrimination is distinguished from stigma which is the cognitive or attitudinal component.

There is also a distinction between discrimination and perceived discrimination, as the two are not synonymous. In order to report perceived discrimination, one has to notice the event, interpret it as discriminatory, and then report it as such. Any of these activities can be interrupted in the process [4,5]. Two competing theories attempt to predict whether minority group members would be more or less susceptible to reported perceived discrimination. The first theory argues that individuals who belong to a minority group, be it ethnic minorities, women, or older people, are more likely to perceive discrimination as they are exposed to a variety of institutional barriers throughout their lives. Supposedly, minority groups are already sensitized to identifying instances of discrimination in their everyday life [6]. An alternative view, however, would argue that being accustomed to differential treatment and attitudes throughout their lifetime results in the internalization of self-stigma [7], such that external acts of discrimination are not necessarily interpreted as such [8]. 

In addition to discrimination by others, be it individuals or institutions, we know that self-stigma or internalized stigma is common among minorities, who accept the negative views of their own group portrayed by the majority culture [7,9]. Similar to the negative effects brought by discrimination imposed by the outside world, self-stigma too can have negative implications for one’s health and well-being [10,11]. It may also result in the justification of a discriminatory situation and may inhibit individuals who experience self-stigma from changing discriminatory situations or even from reporting discrimination [12]. 

On the other side of the spectrum lie the reasons for discrimination. Research conducted in the past few decades showed that attempts to self-enhance, to boost one’s image, to increase a sense of coherence and group affiliation, and to keep the power differential in society motivated discrimination toward other groups [13,14]. Nevertheless, discrimination is not always explicit and may not be driven by a particular motivation. Many times, discrimination can be implicit, automatically triggered by our need for categorization [15].

### 1.1. Perceived Discrimination and the JEWISH–Arab Conflict

Past research described the Arab population in Israel as a society between tradition and modernization [16]. The average level of education of the Arab population is lower than that of Israeli Jews, and their socioeconomic status is also characterized as lower compared with Israeli Jews [17]. Compared with the Jewish population in Israel, older Arabs are more likely to experience functional decline and to require assistance in activities of daily living (ADLs) [18,19]. Moreover, the average lifespan of Arabs in Israel is substantially lower than the average lifespan of Jews, who on average are likely to live three years longer [20]. The reasons for these differences in health status can be attributed to different health habits [21], differential treatment by the healthcare system [21], and perceived discrimination [22].

The Jewish–Arab conflict dates back to over a century ago [23]. The conflict was documented and addressed from a variety of angles [24,25,26], with an eminent question concerning the co-existence of democracy and equal rights to all citizens in a country that defines itself as a Jewish state [27]. In response to this concern, past research showed that Arabs in Israel are quite satisfied with their situation as individuals, but tend to perceive inequalities directed to them as a group by noting both individual and institutional discrimination [26]. Indeed, researchers documented discrimination in the educational system and the labor market [28,29]. Consistently, others addressed discrimination from a political perspective, arguing for the violations of human rights, which are often attributed to security concerns associated with the ongoing conflict [30].

### 1.2. Perceived Discrimination in the Context of the Long-Term Care Insurance Law

The present study adds to this growing body of knowledge by examining perceived discrimination and stigma among Arabs in the context of the Long-Term Care Insurance Law. The National Insurance Institute of Israel (NII) supports formal home care services in the country by providing financial means to older adults who require assistance in activities of daily living (ADLs). This is done through the Long-Term Care Insurance Law, which was issued in 1988 [31]. The support of the law is offered in order to maintain older adults in their homes for as long as possible by providing assistance to family members who are expected to carry the bulk of the care load.

Older Israelis are entitled to a local Israeli care worker for several hours per week (a maximum of 28 paid hours) or a migrant home care worker, who provides live-in services. The number of hours supported by the NII is determined based on the degree of impairment of the older adult and the amount of care required to support him or her in the community. Although there is an upper income criterion over which the NII does not provide financial support to older adults even if they require assistance (approximately 10,000 shekel for a single person and 15,000 shekel for a couple), this bar is relatively high and allows many older adults to receive the support offered by the NII (as the median income in the country stands at a little over 6000 shekel, approximately $1700 United States dollars (USD)) [31].

Currently, 16.6% of all Israelis over the age of 65 receive home care services through the Long-Term Care Insurance Law [32]. In the eyes of the law, the Arab population in Israel is entitled to the exact same services as the Jewish population. Yet, Arabs are over-represented among those who receive the support of the law [33]. This could be attributed to the higher levels of functional impairment found among older Israeli Arabs compared with older Israeli Jews [18] or to the fact that Israeli Arabs are less likely to use institutional care [34]. The majority of older Arabs, who rely on paid home care and receive the support of the Long-Term Care Insurance Law, do so primarily via family members (mainly grandchildren (41%), other relatives (36%), or neighbors and friends (10%)) who are financially compensated for their assistance [34].

To date, there is no research on the perceived or the actual acts of discrimination of the Arab population by long-term care services in Israel. However, we know from research conducted in the United States, as well as in other countries, that ethnic minorities are often quite suspicious toward formal care providers [35,36]. As a result of past negative experience with formal systems and providers, ethnic minorities adopt an approach of suspicion and distrust toward the majority culture. This often results in the underutilization of services and limited cooperation with the formal system [36]. Moreover, past research claimed that the distrust in healthcare providers of the Black population leads to a limited use of preventive services, which, in return, results in deteriorated health among Black older adults [37].

### 1.3. The Present Study

In the light of research conducted in other parts of the world, which documented the impact of perceived discrimination on the health and long-term care service use of minorities and the ongoing tension between Jews and Arabs in Israel, the present study examined the experience of perceived discrimination and stigma of the Arab population in the context of the Long-Term Care Insurance Law. The study was conducted in the north of Israel because this area is heavily populated by Arabs. The Arab population in the north of Israel constitutes the majority, as 53% of the population in the north is Arab as opposed to only 21% of the entire population of the country [17,38]. The north of Israel is somewhat poorer and more rural than the rest of the country [39]. It is less heavily populated than the center of the country, inhabiting only 16% of the population in an area of 4473 square kilometers, compared with the center of the country, in which almost 41% of the population lives [38]. It is also characterized by fewer social services compared with the center of the country. The Arab population in the north of Israel is heterogeneous and is composed of Muslims (39%), Christians (7%), and Druze (8%) [17].

In designing this study, there was no a priori interest in perceived discrimination and, in fact, none of the questions in the interview guide addressed this issue directly. Instead, the larger study focused on intergenerational solidarity in the Arab sector. However, the open nature of the interview guide and the coding scheme allowed for themes that were not pre-conceived prior to embarking on the study to emerge from the data. Given the prominent place the topic of perceived discrimination and stigma received during interviews, perceived discrimination and stigma became the focus of this study. Other themes that emerged during the coding process were delineated in papers on intergenerational solidarity in the Arab community [40] and perceptions of the NII in light of the Long-Term Care Insurance Law [41]. The focus of each paper on a specific core category was done using selective coding, which involves the identification of a core category and related themes which correspond with it [42].

Because the implementation of the Long-Term Care Insurance Law involves multiple stakeholders, including older adults, their family members, and the NII workers who are in charge of implementing the law, all stakeholders were interviewed in this study. Moreover, even though the focus of this paper was on the experience of the Arab population, as an ethnic minority group in Israel, the views of Jews in the country were also examined. The decision to incorporate the views of Jews on the potential experience of discrimination of the Arabs was fueled by the understanding that the two groups are intertwined [43] and that, in order to better understand the experiences of the minority group, one also has to incorporate the experiences of the majority group. This allows for the triangulation of the same core theme, in this case, perceived discrimination and stigma, which is discussed from several different angles. 

Despite the particularities of the law and its existence within the Israeli context, the topic of perceived discrimination and stigma in the context of long-term care can provide important insights into inter-group relationships that go beyond the local context. The opportunity to present the topic from different angles allows for a comprehensive overview of perceived discrimination and sigma as they are described by the minority and the majority group, as well as by consumers and service providers. As research on perceived discrimination and stigma in the context of long-term care is scarce [44,45], this study provides an important perspective that received only limited attention thus far.

## 2. Methods

### 2.1. Design

This study was conducted using thematic analysis to identify patterns in the data. The advantage of the method stems from the fact that it is not limited by a certain epistemological or theoretical perspective. In this study, there was no prior guiding theory. Instead, the analysis focused on identifying themes, with the realization that we cannot bring the voice of the interviewer as is, but instead interpret the findings to reflect meaningful patterns in the data. In analyzing the data, we moved from semantic themes, which simply describe the data, to latent themes, which attempt to provide meaning to the data [46].

### 2.2. Sample

The study was supported by a grant from the NII and approved by the ethics committee of the principal investigator (PI)’s university. In order to obtain a broad overview of the topic from the perspective of multiple stakeholders, we interviewed all individuals involved in the Long-Term Care Insurance Law. This includes NII workers in charge of implementing the law, older adults, their family members, and paid home care workers. Although the focus of this study was on the Arab population, we also incorporated the point of view of the Jewish population, as this provides a complimentary view of the majority culture on the experiences of the minority group. 

In selecting participants, we specifically selected individuals who worked (e.g., NII workers) or resided (e.g., older adults) in different geographic regions in the north of Israel (e.g., mixed cities of both Jews and Arabs, solely Arab or Jewish cities, and small villages). The socioeconomic status of these areas varied between two and five, out of a maximum of 10, according to the Israeli Central Bureau of Statistics. Hence, they all tended to be on the lower side of the socioeconomic spectrum. The distance to NII services also differed, with some areas having the NII facility in close proximity and others requiring a commute in order to reach services.

The NII provided the research team with a list of workers who worked in the north of Israel at the time of the study and were either responsible for (a) the overall management of the home care unit (e.g., managers), (b) the evaluation of older adults’ needs and functional disability (e.g., evaluators), or (c) the determination of the level of financial support that should be provided to the older adults (e.g., consultants). The NII also supplied the research team with a list of names of older adults over the age of 70 who applied for financial assistance through the Long-Term Care Insurance Law and resided in the north of Israel. These individuals were firstly contacted via mail by the NII, and those who did not express an explicit refusal were invited to participate in the study. We interviewed participants regardless of whether or not their claim to the NII was successful. We approached these individuals and asked for the contact information of their main family caregiver and paid home care worker, when applicable. These individuals were also invited to participate in the study. Overall, we interviewed 15 National Insurance Institute workers (NII; responsible for implementing the law; 47% Arab), 31 older adults (81% Arab), 31 family members (87% Arab), and six paid home care workers (83% Arab). The details of the sample are listed in Table 1.

### 2.3. Data Collection

The interview guide was constructed as a funnel. NII workers were asked to describe their role and their relationships with the various stakeholders: older adults, family members, and home care workers. They were asked to detail the strengths and weaknesses of their role, as well as their coping mechanisms with various challenges. Finally, they were asked to describe and contrast their encounters with the Jewish and the Arab populations and to potentially identify differences between the two population groups. They were also asked about the perceived uniqueness of the north of Israel compared with other regions in the country.

Interviews with older adults, their family members, and paid care workers started with broad questions concerning the home care arrangement and relationship between the involved stakeholders (e.g., older adults, family members, paid carers) in the beginning of the interview and more specific questions toward the end of the interview guide. Broad questions included the following: “tell me about your relationship with other family members/the older adult/the home care worker” and “tell me about the type of care you receive/provide”. Respondents were asked comparative questions, such as “what is the difference between the care provided by a paid home care worker and the care provided by a family member”, “what is the difference between care provided in the Arab sector vs. care provided in the Jewish sector”, and “what is the difference between elder care provided in the north of Israel vs. other parts of the country”. Respondents were asked descriptive questions, such as “how did you reach the decision to approach the NII” and “tell me about your encounter with the NII officers”. They were also asked interpretive questions, such as “what are the challenges of having a paid home care worker” or “what is unique about the care provided in the north of Israel”. See Appendix A for the interview guide.

Interviews lasted between 30 minutes and one hour. Interviews were conducted in the place of choice of respondents. All interviews were recorded and transcribed verbatim. Interviews were conducted in Hebrew or Arabic to meet the preference of respondents. Interviews conducted in Arabic were translated into Hebrew. Interviews with the Arab population were conducted by Arab interviewees, and interviews with the Jewish population were conducted by Jewish interviewees. Interviewees were undergraduate or graduate students in the field of social science. All interviewees had prior training in qualitative research. They also received specific training for the purpose of this study, which was administered by the PI (a psychologist with more than a decade of experience in qualitative research).

### 2.4. Analysis

Interviews were analyzed thematically, using Braun and Clarke’s (2006) six-stage method. This approach was extensively used in research conducted in long-term care [47,48,49]. I started by approaching the interviews with no pre-conceived aims, using open-coding of the smallest units of meaning in the text [50]. Each interview was analyzed separately at this stage. To do so, I generated initial codes, such as “good care”, “the benefits of paid care”, or “loneliness”. Subsequently, codes were collapsed into larger categories of meaning (e.g., themes), while comparing and contrasting within and across interviews. Excerpts from the interviews were entered into a table that included the major themes or patterns of meaning identified. Comparisons and contrasts were conducted within and between interviews to collapse smaller units of meaning into larger themes [51]. For instance, a code called “perceived discrimination” included smaller codes, which indicated a sense of unfair treatment due to the respondent being an Arab minority in the country, such as “the NII always refuses”, “the NII is slow on purpose”, or “the NII treats Jews and Arabs differently” (see Table 2 as an example). At this stage, each of the interviews was reviewed once again. Next, I used selective coding to create a coherent storyline [42]. Of the various themes identified, I selected themes that addressed the concepts of discrimination or internalized stigma. These themes were selected because of their prominence in interviews conducted with the Arab population. These themes are described in detail in the findings section. For triangulation, each of the themes was examined from the perspective of the various stakeholders who were interviewed in this study. Comparisons and contrasts between stakeholders are outlined. 

### 2.5. Sources of trustworthiness

The interviews were conducted with four different stakeholders to provide a broader outlook on the phenomenon. Moreover, we integrated the point of view of the Arab population with that of the Jewish population in order to obtain a more comprehensive outlook on the topic. All stages of data collection and analysis were fully documented to provide an audit trail [52]. In addition, a thick description was provided by suppling detailed quotes from the interviews to allow the readers an opportunity to judge the adequacy of the categories proposed [53]. Finally, the coding scheme was discussed with policy-makers in the NII to obtain their feedback. This is one form of member check, which adds credibility to the analysis. Whereas some NII employees were quite eager to learn more about the topic and even suggested future research to examine the topic in more detail, others negated the findings and adamantly stated that there was no discrimination in the allocation of the Long-Term Care Insurance Law.

## 3. Results

Four major themes were identified. The first theme concerned a very strong sense of discrimination and disentitlement perceived by the Arab interviewees as being directed toward them by NII policies and employees. Almost all Arab interviewees were very confident about the superior care provided to the older Jewish population in the country. Furthermore, the most common explanation attributed to the NII declining their claims to obtain the support of the Long-Term Care Insurance Law was perceived discrimination directed by the country of Israel toward its Arab citizens. A second theme addressed internalized stigma by Arabs; both NII workers and very few Arab older adults and family members argued that the Arab population prefers to be served by Jewish NII workers as they are seen as providing more adequate services. A third theme, which can be described as implicit racism, argued that (primarily) the Arab population is “cheating the NII system” and submitting false claims for support. Finally, a fourth theme stressed by NII workers emphasized the balanced and even-handed approach toward Arabs by the NII. The four themes are detailed below, accompanied by supporting excerpts from the interviews. These four themes clearly demonstrate how the affiliation with the minority vs. majority group makes a major difference in one’s perceptions of discrimination and stigma in the context of the Long-Term Care Insurance Law.

### 3.1. Perceived Discrimination

A major theme almost unanimously agreed upon by Arab interviewees, whether they were older care recipients, their family members, or their paid home care workers, was the strong sense of perceived discrimination as a result of their Arab origin. Interviewees argued that, even without knowing the other side, e.g., Jews, they are confident that they receive poorer services or even inadequate services as a result of their population group affiliation. The following is a quote by a 30-year-old Muslim home care worker: “The Jews have more rights; they receive more things. Older adults in the Jewish sector receive more. For instance, I see older adults, who are completely healthy and can serve themselves. Nevertheless, they receive paid care just because they live on their own. Here, there are many Arabs who live alone, but are not entitled to receive the Long-Term Care Insurance Law. It is clear that there is discrimination.”

Even those interviewees who acknowledged having no concrete information about the state of Jews in the country still reported a strong sense of discrimination, which according to them shapes the services provided to older Arabs: “I have no knowledge or experience with regard to the Jewish society. I assume that their situation is better. But I really do not know. I do not know the situation over there. I know they receive more rights. They receive more with regard to everything. This I know in general. More than this, I do not know,” a 59-year-old Druze, daughter of an older care recipient.

Whereas some interviewees were very clear that discrimination was due to pure racism, others attempted to attribute it to other reasons, such as proximity to the older adult or to the better care provided by family members in the Arab sector. The following is a quote by a 52-year-old Christian daughter: “Maybe the difference is that, here, Arabs live in close proximity. Family members live nearby. The daughter, the daughter in law, the son, everyone is nearby. Their (Jewish older adults) situation is different. Everyone lives in different places. Different cities. One lives in the center and one in the south. Unlike us, when the son lives above the parents or next to them. Maybe this is why they (the NII) are quick to approve long-term care assistance to older Jews. Here, among the Arabs, they are more likely to trust family members. They abuse the fact that we live nearby, so they provide us with fewer services.” 

Nonetheless, many attributed the denial of their claim for support to pure discrimination. Respondents argued that, had they been Jews, their application would have been approved and they would have received financial support through the Long-Term Care Insurance Law. The following is a quote by a 77-year-old Muslim care recipient: “Here, there is discrimination. It is their (Jews) country. They do not care about the Arabs. We pay social security even more than the Jews. They go to the army and receive many things. Every older Jew immediately receives all the rights. Here (in the Arab sector), you need to fight over everything. Here, there are many refusals. Even if they see you in a wheelchair, they do not take this into account. I tell you explicitly: there is racism here and this country does not treat the Arabs well.”

A similar message was conveyed by a 73-year-old Muslim care recipient: “With us, an NII worker comes in order to fail you, not in order to help you. With Jews, the worker comes to help and he actually helps.”

It is interesting to contrast this confidence in the experience of discrimination based on population group affiliation with the uncertainty reported with regard to the state of services provided in other geographic regions in the country. Whereas most Arab interviewees clearly acknowledged (with no explicit prompting) the presence of discrimination due to race, they were unclear about the perceived presence of regional differences. The following interview with a 78-year-old Muslim care recipient demonstrates this:

Interviewer: “What do you think are some of the differences between the care provided to older Jews vs. older Arabs in the country?”

Interviewee: “There is a lot of discrimination. This is a political affair. Differences exist in all spheres of life.”

Interviewer: “What are the differences in the care provided to older adults in the north of Israel vs. other areas such as the center or south of the country?”

Interviewee: “I have no idea. I have been receiving long-term care support only for the past two years. I really have no idea what happens in other areas.”

### 3.2. Internalized Stigma

Whereas the experience of perceived discrimination was widely acknowledged by most Arab respondents, the report of internalized stigma was not common in this population. However, a few interviewees reported a strong preference toward and interest in “crossing lines” and joining the other side, namely, the Jews in the country. The following is a quote from a 47-year-old Muslim son: “I am ashamed. I tell you, only because of the shame and my respect. Otherwise, I would have converted to Judaism a long time ago. They have more mercy and more help. They took the good things we had and we took from them only the bad things.” 

A 44-year-old Muslim daughter also regarded the working conditions as being preferable under a Jewish employer: “I know workers who care for older adults. They are happy. They receive more. It is different. The attitude over there is different. Salary is higher, benefits are better.” 

Another form of internalized stigma was evident in reports of Arab family members and older adults concerning the disqualification of Arab NII workers compared with Jewish workers. This account was reported within the overall context of a strong sense of perceived discrimination, arguing that discrimination is particularly pronounced when it comes from Arabs toward their own people: “It is due to the neglect of the Arab officer. The Arab officer neglects his work. Does not provide services as required to the Arabs here. My wife is sick, lying in bed. I am sick too. My daughter in law should not be coming here to help and care. She has kids, a husband, and she has two kids in a poor condition. How much can she suffer?” It is important to note that this is not to say that this interviewee is happy with the Jewish workers at the NII, but the Arab workers seem even more hostile and discriminatory in her view.

These views were augmented in interviews with the NII workers, who specifically argued that the Arab population prefers to be evaluated by Jewish workers. The NII workers also said that Jews also prefer to be seen by a Jewish worker. Hence, they argued that an Arab NII worker would suffer from stigma, associated with being Arab when serving the Arab community, as well as when serving the Jewish community: “There is something really interesting. We send Arab speaking evaluators to assess Arab families, but they want Jews for some reason. And I tell them, ‘we have to send someone who speaks the language. Who understands the older adult.’ But, they tell me, ‘send the Jews’. The opposite direction almost never happens. We had one (Jew) who asked not to get the ‘racist Arab’. There was this period of terrorist attacks, so we did not send Arabs to Jewish families because there was a lot of tension,” a Jewish NII manger.

### 3.3. Implicit Stigma: (Mainly) Arabs Are “Cheating” the System

Despite the fact that most NII workers regarded claims about discrimination as unsupported, many reported a strong suspicion that Arabs more so than Jews try to mislead the system in order to receive unjustified financial assistance: “When you go to a family as a person who represents the NII, the approach of the Arab society to authority is to use as much as you can and get as much as you can. It does not matter whether or not you deserve. The approach is always negative. They see you as representing authority and they also approach you negatively. This creates a challenge. I spoke earlier about unreliability. This is the result. They do everything to justify the fact that they deserve assistance. Especially the Druze. They say, ‘I deserve, I served my country. So I deserve. This is a privilege which I deserve even without checking’,” a Muslim NII evaluator.

Abuse of the system and unreliable reports were also attributed to the poor financial situation of the Arab population. Accordingly, for many families, the assistance provided by the NII is seen as necessary for their survival: “There is a financial difference, income difference, between the Arab and the Jewish sectors. The two sectors are very different financially. These differences make Arabs seek the financial support of the NII much more than Jews would,” a Muslim manager.

Other reasons for not providing reliable information to the NII evaluators were a lack of knowledge and attempts of private home care agencies to manipulate families and instruct them on how to submit unjustified claims for home care support. The following statements were made by a Jewish agency manager: “Sometimes, they (home care agencies) manipulate a geographic area, an entire sector. There is really aggressive activity. It is really a problem it creates pressure on the system. Claims that were submitted two months ago, and there was no worsening of the situation yet, are being resubmitted again and again and you know they will be denied. We come without setting an appointment and see the house is clean, the woman is clean, getting out of the house. And then she sees us, ‘give me a cane.’ There also are companies that prepare the older adults. They place diapers that smell of urine. Do not ask how far this has gone.”

The notion of cheating the system was brought up infrequently in interviews with older adults and their family members: “Once a woman from the NII came over. A Yamani Jew. She wanted to see how I function in the kitchen. My daughter screamed at me to drop things off my hands. I immediately screamed back that these workers are not stupid. They see everything. Then they (NII) said I do not deserve support. I can still do things,” a 75-year-old Muslim care recipient. 

### 3.4. There is No Discrimination

The strong sense of perceived discrimination was contrasted with the reports of the Jewish interviewees. When Jewish older adults and family members were asked to compare the care provided to older adults in the Jewish community to the care provided in the Arab community, they explicitly argued for no evidence of discrimination and for no preferred treatment of older Jews: “I have no connection. I do not have much contact with Arabs. But, I understand with Arabs you have to be like people, like with the Jews. They have their religion. We have our religion. There is no difference. We live here together. We will continue to live here together. No difference between us.”

A similar argument was made by only very few Arab interviewees: “No. I do not think there are any differences. Everyone is entitled to submit a request for financial support through the Long-Term Care Insurance Law and everyone receives assistance based on his or her condition,” a Muslim home care worker. 

Arguments concerning no differences between the two sectors were strengthened through the report of the NII workers, who all stressed the equal treatment provided by the NII, unrelated to race or religion. The following is a quote of a Muslim evaluator: “There is no substantial difference between a home visit to see an Arab or a Jew in my opinion. It is the same thing in my mind. That I am in a house of an Arab or a Jew is the same for me. I am seen with the same level of warmth by both sides. Sometimes, in the same level of ambivalence. The fact that you talk about a Jew or an Arab does not create a difference. It could be an angry Arab and an angry Ashkenazi Jew. The fact that it is a Jew or an Arab does not make a difference.”

Another Muslim NII evaluator provided a similar rationale: “My approach is the same approach. Every person receives services unrelated to his or her religious background or nationality. My concern is that this person will receive the best assessment and the right services he or she needs and receive the financial assistance he or she needs. There is no discrimination toward people, whether they are Jews or Arabs. There is no preference of Arabs over Jews. I do not give a preferential treatment to Arabs because they are Arabs and no preferential treatment to Jews because of other reasons.”

## 4. Discussion

The present study examined the perceived discrimination and stigma of the Arab population in the north of Israel in the light of two competing theories. One theory would argue for a sensitizing effect of past experiences which make the Arab population more susceptible to report the experience of discrimination. Another theory, in contrast, would argue for an opposite prediction, arguing that individuals of a minority status already internalize past experiences of discrimination and, therefore, are less likely to report current experiences of discrimination. The present study shows that both hypotheses have some merit. The Arab population was more likely to report perceived discrimination, even if they did not experience it in the specific context addressed in this study. However, a portion of the Arabs interviewed in this study also reported self-stigma. Hence, both theories received some support in this study.

This study addressed the topic of perceived discrimination and stigma from the eyes of Arab older adults, family members, and paid home care workers. Their perspectives were complimented by views of Jewish older adults and Jewish family members, as well as by the reports of Arab and Jewish NII workers. Such a 360-degree study allowed for a more comprehensive appreciation of the power differences between Jews and Arabs in the country, as well as between older adults, family members, home care workers, and NII workers. 

The study did not aim to assess the objective state of Arabs in the country, but rather their subjective experiences and perceptions. In reviewing the findings, it is important to stress the fact that the topic addressed in this paper spontaneously emerged from interviews with the various stakeholders. Moreover, even though unsolicited, this sense of perceived discrimination was the most consistent finding across interviews with all stakeholders. 

A major finding of the present study concerns the very salient presence of discrimination in the life of the Arab population, as interviewees openly and without further prompting discussed their perceived experience of discrimination. The sense of discrimination among Arabs is not new, as past research showed perceived and actual discrimination of this population group in multiple settings. For instance, a recent study showed that the majority of Jewish customers prefer to receive services from firms that employ Jews, rather than Arabs. This, in return, affects the firms’ hiring decisions. Moreover, those firms that employ Arabs charge significantly lower service prices [54]. A different study showed that Jewish landlords are 2.4 times more likely to confirm the availability of property when the caller is Jewish rather than an Arab potential renter [55]. Given past research, which showed a direct association between perceived discrimination and multiple negative outcomes, including higher levels of depressive symptoms [56], smoking [22], and poorer health [57], addressing sense of discrimination in this population is highly important. 

This sense of perceived discrimination was internalized by some of the interviewees, with some reporting negative views toward their own reference group. Opposite opinions, however, were expressed by NII workers, who implicitly reported stereotypical thinking toward the Arab population, viewing it as if it were “cheating the system”. Moreover, the Jewish population interviewed in this study adamantly negated any discrimination toward Arabs. These findings contribute to our understanding of intergroup relationships by pointing to the important role of the perceiver: minority vs. majority group, care recipient vs. care provider. 

The study has major implications for the fragile political situation in Israel. First and foremost, it shows that a strong sense of discrimination exists and that the minority group in society believes that it received inferior services compared with the majority group. The majority of Arab older adults, family members, and home care workers explicitly stated that they were being discriminated against. Although they were only queried indirectly about their home care experience in the Arab society compared with that in the Jewish society, the first thing that came to their mind was the fact that they are discriminated all their life in all spheres of life. Apparently, they do not need to experience discrimination in order to “know” that it exists. To some degree, this is an expected finding given the breadth and depth of the Arab–Israeli conflict [58]. However, it is unexpected and alarming if, indeed, the perceived sense of discrimination reported by Arab consumers is based on inadequate treatment provided to the Arab population by NII workers. To the best of our knowledge, this is the first study to document this sense of discrimination by giving a voice to people to openly express their grievances in the context of elder care. To better test whether, indeed, these feelings are based on objective reality, further research, which compares objective evaluations done by the research team to the evaluations done by the NII, is needed. To the best of our knowledge, such analysis is yet to be conducted. 

There is ample literature on the challenges faced by ethnic minorities in the use of formal health services [37,59]. Suspicious, distrust, and underuse are common results of past experiences of discrimination. The present study further suggests that, perhaps in the Israeli case, attempts for overuse for monetary benefits by submitting multiple claims are also a result of perceived discrimination. As a result of a strong sense of discrimination, Arab older adults and their family members might submit a large number of claims to the NII following the rationale that the system ignores their needs and rights. Many believe that, no matter what the situation is, they will not receive adequate treatment. This strong conviction in their exposure to discrimination was evident even among those individuals who admitted to not having experienced discrimination themselves. Nonetheless, they just “knew” it existed.

Perceived discrimination is subjective. In order to acknowledge discrimination, one has to notice an event, interpret it as discriminatory, and then report it. Any of these processes can be affected by a number of factors [4]. The present study clearly shows how the perspective on whether or not discrimination exists varies depending on who provides the information. Arabs, whether they were older adults, family members, or home care workers, acknowledged the experience of discrimination by the majority culture, namely, the NII. Jewish interviewees, on the other hand, denied the existence of discrimination all together. As the ones who represent the majority culture, it is probably a “safer choice” not to acknowledge discrimination toward minorities in the country. Similar to Jewish interviewees, NII workers, whether they were Arab or Jewish, adamantly denied the experience of discrimination toward Arabs. NII workers also represent the majority culture, unrelated to their religion. They work for an official governmental organization, and a non-discriminatory approach is equated with adequate services. 

The NII workers’ attribution of dishonesty to the Arab population and the tendency to blame primarily (but not only) the Arab population in the submission of multiple false claims could be seen as an attempt to justify discriminatory behaviors and attitudes toward the Arab population. Interestingly, many of these claims came from Arab NII workers, who potentially identified with the majority group and internalized a sense of self-stigma. It is also possible, however, that the Arab population in Israel does submit a large number of claims due to financial difficulties faced by the Arab sector in Israel [17] and the view of the law as a way to obtain financial assistance, unrelated to their physical status. 

Another reason for this behavior could be attributed to practices of private home care agencies in the county that, reportedly, tend to encourage older adults to submit claims (as reported by NII workers). Although the support to employ a home care worker is provided by the government through the Long-Term Care Insurance Law, the actual provision of services is outsourced to private agencies that assist in matching workers to families for profit. In such an arrangement, the agencies have an incentive to submit as many possible claims as possible, with the hope of getting at least some of the claims funded through the Long-Term Care Insurance Law [40]. Under such circumstances, less educated and knowledgeable older adults and family members might be more likely to follow these bogus seductions and submit false claims. Finally, it is possible that the view of many Arabs of the NII policy as representing the Jewish/Israeli policy of discrimination and the provision of limited rights and benefits to its minority citizens encourages a behavior that is more lenient toward the submission of unjustified claims. Further quantitative research is required in order to compare Arabs and Jews with regard to the type and number of claims submitted to the NII versus the actual approval of these claims. Such a study should obtain independent evaluations of the claims and compare them against the NII evaluations in order to determine whether or not discrimination exists. Nevertheless, the sense of perceived discrimination is likely just as important as whether or not discrimination actually occurs and, as such, should be regarded seriously by policy stakeholders. 

The present study further shows that, similar to other “isms” [9,12], some Arab interviewees internalized the views of the majority culture. As a result, they did not want to have Arabs as NII evaluators, arguing against their fair judgement. Others explicitly expressed a wish to become Jewish in order to enjoy the same level of services. Yet, others (Arab NII workers) stated that Arabs are cheating the system and are unreliable in the information they provide to the NII. These are various manifestations of a similar phenomenon, which represent the internalization of negative views toward one’s group, possibly due to the inferior position of this group in society.

Of note is the finding that both the Jewish population and the NII workers adamantly negated any discrimination toward Arabs. Past research stressed the significant role of micro-aggressions in everyday life, which are often not noticed by the majority culture [60]. The present study elaborates past research by showing that the negation of discrimination is done not only in everyday life, but also with regard to major life events, such as the Long-Term Care Insurance Law. Both groups interviewed in this study have an incentive not to acknowledge discrimination. The NII workers have an incentive to negate any form of discrimination, as unequal treatment suggests inadequate performance on their part. Similarly, the Jewish population potentially also has an incentive to negate unfair treatment, as its presence reflects badly on the services they receive and present them as enjoying benefits at the expense of others. This research potentially provides support for the automaticity of discrimination, rather than as a means to enhance self-esteem or achieve personal goals [15].

### Limitations

This study should be viewed in light of its limitations. Firstly, even though it is not uncommon and, in fact, could be seen as a strength of qualitative research, the topics discussed in this paper were not pre-conceived a priori, and this research represents a bottom-up approach to data analysis and collection. Future research will benefit from developing a more systematic approach to the topic to explore the potential manifestations of perceived vs. actual discrimination in the context of home care services. A second limitation concerns the sample. Even though we attempted to obtain maximum variations, we have no representation of some minority groups in Israel such as the Bedouin population and have very limited representation of Druze. We also have limited representation of paid home care workers, as most families interviewed had no such care. This means that most of our interviewees applied to the NII in order to obtain financial assistance, but their claim was denied. Hence, we interviewed a highly selective and probably disappointed sample. Moreover, we ended up interviewing approximately 25% of the sample which was provided to us by the NII. Reasons for not interviewing participants were refusal to participate, no command of Hebrew or Arabic, inadequate contact information provided by the NII, and death or sickness of the older care recipient. Finally, it is important to acknowledge the subjective nature of this study. There is certainly room for subjectivity, especially when the topic of perceived discrimination is addressed. Nevertheless, these subjective reports are uncorroborated by actual data on claims and their approval or disapproval in the different sectors. Future research comparing the NII evaluations to evaluations conducted by the research team is desired. 

## 5. Conclusions

Despite these limitations, the present study sheds a new light on an unexplored topic, which likely has major implications not only in the field of home care services, but for the ongoing relationship of Arabs and Jews in the county. Our findings show that a sense of perceived discrimination is common and colors the experience of service seeking and service satisfaction among Arabs. These findings likely have major implications for both service providers and policy-makers and legislators. Theoretically, this study shows that the Arab minority in the country tends to report higher levels of discrimination, but, at the same time, also internalizes negative views toward their own group.

The findings can inform policy stakeholders on how to better serve the Arab community in Israel. Even if perceived discrimination does not reflect objective discrimination, interventions should still attempt to target the strong sense of perceived inferior unjust treatment reported by this population group. Globally, the findings add to a better understanding of the fragile position of ethnic minority older adults who often are affected by a double jeopardy due to their old age and ethnic minority status. Although past research showed that a common reaction to perceived discrimination is reduced levels of service use, this study shows that, when service use is associated with financial benefits, perceived discrimination may result in higher levels of use. 

Future research will benefit from objectively examining the claims made by Arab and Jewish older adults to assess for differential treatment between the two population groups. At this point, the study may raise potential hypotheses concerning the sense of perceived discrimination reported in this study that should be further examined in future research. For instance, it is possible that Arabs have high expectations for the type of services and benefits that they should get and that, if these expectations are not met, the Arab population is misinformed about its’ rights. It also is possible that cultural barriers prevent Arab clients from better understanding the Long-Term Care Insurance Law criteria. Additional research might also benefit from focusing on subtypes of respondents to identify additional variables that contribute to the perception of perceived discrimination within the Arab group as some variability in responses was noted. 

## Figures and Tables

**Table 1:** Demographic characteristics of the sample.

	NII Workers (*N* = 15)	Older Adults (*N* = 31)	Family Members (*N* = 31)	Home Care Workers (*N* = 6)
Age in years	46 (8.1)	79 (4.6)	51 (12.5)	35 (16.0)
Men	7	7	15	1
Education in years	16.2 (1.2)	5 (4.4)	11 (4.4)	10 (3.9)
Jews	8	6	4	1
Christian	1	2	2	
Druze	1	1	2	
Muslim	5	22	23	5
Relationship to the older adults				
Child	NA	NA	16	NA
Spouse			5	
Other			10	
Weekly number of hours of care	NA	NA	10 (6.5)	NA
Daily number of hours of care and number of days care is provided	NA	NA	NA	3 (1.0) and 5 (1.6)
Managers	9	NA	NA	NA
Evaluators/consultants	6			
Subjective health (1–5)	NA	1.9 (0.6)	3.6 (0.9)	3.8 (0.4)
Subjective socioeconomic status (1–4)	NA	1.7 (0.7)	2 (0.6)	2 (0.6)
Have a home care worker	NA	15	NA	NA
Yes				

NII, National Insurance Institute; NA, not applicable.

**Table 2:**
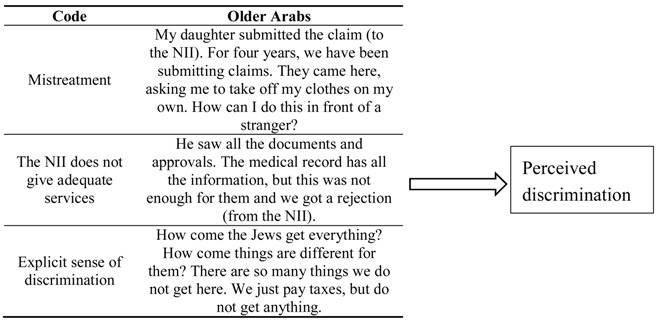
An example of the coding scheme used in the present study and the transition from codes to themes.

## Appendix A. Interview guide

Older adults and famliy members were asked the following questions:

- Tell me about your relationship with family members/older care recipient/paid home care workers.
- Tell me about the care you receive/provide.
- What are the differences between paid/unpaid/family care? What are the similarities?
- What are the advantages/disadvantages of each paid/unpaid/family care?
- What is unique to care in the Arab/Jewish sectors? What are the differences in care between the two sectors?
- Tell me about the decision to ask for long-term care insurance.
- How was your encounter with the National Insurance Institute?
- What are some of the challenges? What helped you in dealing with the institute?
- If your claim was rejected—how do you explain this? Why do you think your claim was rejected?

Home care workers were also asked the following additional questions:

- Tell me about your work.
- Why did you pursue this job? Why older adults?
- Workers of the National Insurance Institute of Israel were asked the following questions:
- Tell me about your job.
- Tell me about your relationship with paid workers—foreign, Arab? With family members? With older adults?
- What makes you feel satisfied at work?
- What are some of the challenges? The difficult parts at work?
- What are the strengths and weaknesses of this area? What is unique about this area? How is it different from other areas?
- Tell me about your work with the Arab population? How is it different from your work with the Jewish population?
